# Impact of Comorbidities and Smoking on the Outcome in Aneurysmal Subarachnoid Hemorrhage

**DOI:** 10.1038/s41598-018-30878-9

**Published:** 2018-08-17

**Authors:** Alexander Hammer, Anahi Steiner, Gholamreza Ranaie, Eduard Yakubov, Frank Erbguth, Christian M. Hammer, Monika Killer-Oberpfalzer, Hans Steiner, Hendrik Janssen

**Affiliations:** 1 Department of Neurosurgery, Paracelsus Medical University, Breslauer Str. 201, 90471 Nuremberg, Bavaria Germany; 2 Department of Neurology, Paracelsus Medical University, Breslauer Str. 201, 90471 Nuremberg, Bavaria Germany; 3 Department of Anatomy 2, University of Erlangen-Nuremberg, Universitätsstraße 19, 91054 Erlangen, Bavaria Germany; 4 Paracelsus Medical University, Neurology/Research Institute of Neurointervention, Ignaz Harrer Str. 79, Salzburg, Austria; 5 Department of Neuroradiology, Nuremberg General Hospital, Breslauer Straße 201, 90471 Nuremberg, Bavaria Germany

## Abstract

The intention of this observational study is to show the significant impact of comorbidities and smoking on the outcome in aneurysmal subarachnoid hemorrhage (SAH). During this observational study 203 cases of treatment of ruptured intracranial aneurysms were analyzed. We examined and classified prospectively the 12 month outcome according to the modified Rankin Scale (mRS) considering retrospectively a history of smoking and investigated prospectively the occurrence of early and delayed cerebral ischemia between 2012 and 2017. Using logistic regression methods, we revealed smoking (odds ratio 0.21; p = 0.0031) and hypertension (odds ratio 0.18; p = 0.0019) to be predictors for a good clinical outcome (mRS 0–2). Age (odds ratio 1.05; p = 0.0092), WFNS Grade (odds ratio 6.28; p < 0.0001), early cerebral ischemia (ECI) (odds ratio 10.06; p < 0.00032) and delayed cerebral ischemia (DCI) (odds ratio 4.03; p = 0.017) were detected as predictors for a poor clinical outcome. Significant associations of occurrence of death with hypertension (odds ratio 0.12; p < 0.0001), smoking (odds ratio 0.31; p = 0.048), WFNS grade (odds ratio 3.23; p < 0.0001) and age (odds ratio 1.09; p < 0.0001), but not with ECI (p = 0.29) and DCI (p = 0.62) were found. Smoking and hypertension seem to be predictors for a good clinical outcome after aneurysmal SAH.

## Introduction

With an overall incidence of 9 per 100000 person – years subarachnoid hemorrhage (SAH) is a rare but severe form of stroke^1^. Aneurysmal SAH represents approximately 5% of all strokes and is therefore an uncommon cause of stroke mortality, but occurs at a young age leading to premature mortality comparable to ischemic stroke^1,2^. Interestingly a decline in the incidence of SAH was observed over the last decades^1,3^. Some authors assume associations of the decreasing incidence of SAH with decreasing smoking rates over time, as smoking, hypertension and alcohol abuse are recognized to be important risk factors for SAH^3,4^. Female sex is also confirmed to be associated with a higher risk of SAH. Also heavy smoking is a dose dependent risk in females^5,6^. Ethnical aspects seem to be risk factors while the relevance of hypercholesterolemia, and diabetes remain unclear, although there is increasing evidence that hypercholesterolemia increases the risk of SAH^4,5,7^.

Regarding clinical outcome after aneurysmal SAH demographical parameters and clinical presentation such as age, neurological grade, and aneurysm size seem to play important roles^8^.

Considering the effect of comorbidities and life style risk factors on the outcome of SAH, particularly the impact of smoking and hypertension remain unclear, and published results are contradictory^9–12^. On the one hand smoking was described to be associated with delayed neurological deterioration but without proof of downgrading the clinical outcome^9^. On the other hand smoking is reported to have a protective effect regarding the risk of death in aneurysmal SAH^10^. In the same study hypertension had no effect on the risk of mortality^10^. In a recently published study, smoking was associated with superior outcome compared with nonsmokers^11^.

In this observational study we focused on the evaluation of the impact of comorbidities and life style risks on the outcome of aneurysmal SAH. We particularly focused on the association of smoking and hypertension with the clinical outcome after aneurysmal SAH.

## Methods

We analyzed 203 cases of ruptured intracranial aneurysms with subsequent subarachnoid hemorrhage from 2012 to 2017. Primary endpoint was the clinical outcome after one year and occurrence of ECI/DCI were secondary end-points. Specialized vascular neurosurgeons and endovascular specialists ensured expert treatment in this observational study. Over the study period neurosurgical treatments were performed at the responsibility of 4 neurosurgeons and endovascular procedures were performed at the responsibility of 3 neuroradiologists. The decision of neuroradiologists and neurosurgeons as to the allocation of the patients to the endovascular or microsurgical treatment branch was part of the standard care of the patients. Endovascular procedures comprised sole coiling, coiling in combination with balloon or stent assisted remodeling or the use of endovascular or intrasaccular flow-diverters.

Criteria for study inclusion were:

1. Time between aneurysm rupture and treatment <48 hours
2. Informed consent from the patient, a patient’s relative or the patient’s guardian
3. Verification of SAH with cranial CT or lumbar puncture and verification of an associated intracranial aneurysm diagnosed in most cases by digital subtraction angiography, alternatively by CT angiography if an immediate operation had to be performed.
4. Patient survival until completion of aneurysm treatment.

During stay in hospital we examined prospectively the appearance of early cerebral ischemia (ECI) and delayed cerebral ischemia (DCI). Early cerebral ischemia was defined as a clinically apparent new ischemia within the first 3 days after treatment, detectable in diagnostic imaging. All events that occurred 3 or more days after treatment were defined as DCI. Definition of DCI includes a focal (hemiparesis, aphasia, hemianopia, or neglect) or global neurological impairment lasting for at least 1 hour and/or cerebral infarction, which is not apparent immediately after aneurysm treatment and that cannot be attributed to other causes^13^.

Within the prospective data acquisition of this study 6 and 12 month follow-up telephone interviews were established in order to evaluate the patients’ clinical outcome using the modified Rankin Scale (mRS). The final 12 month telephone interviews were conducted within one month after one year after the initial SAH. The study was approved by the local review board (Ethik-Kommission der Bayerischen Landesärztekammer (2017-133 fm/Gu)) and all research was performed in accordance with relevant guidelines/regulations. Informed consent of the patients or their relatives was obtained during the initial hospital stay or during the telephone interview in the follow up. In total for determination of ECI and DCI all 203 patients were available. Outcome data were available for 199 of 203 patients.

We collected and analyzed all available data concerning pre-defined comorbidities and history of smoking. This data was surveyed on patients admission and was documented in the patients file. But analysis of this data for the purpose of this study was done retrospectively. Smoking was only counted if patients were current smokers. If smoking was quit in the past patients were not considered as smokers. For this purpose all available medical records were analyzed. We screened for the following comorbidities: Stroke, coronary heart disease, atrial fibrillation, hypertension, renal insufficiency, diabetes mellitus, history of ethanol abuse, history of tumor, hypothyroidism, depression, hypercholesterolemia, migraine and history of smoking.

With the available baseline data, comorbidities, history of smoking, ECI and DCI, we analyzed potential protective or predictive effects regarding a poor clinical outcome (mRS ≥ 3) and the occurrence of death after one year. Further we analyzed the influence of demographical data, clinical presentation, the comorbidities and history of smoking on the occurrence of DCI. We included the appearance of vasospasm detected by transcranial Doppler (TCD) (mean flow velocities ≥120 cm/sec) during the intensive care stay.

Data of this study were partially published previously under different aspects by our group^14,15^.

### Statistics

We performed the descriptive statistics (frequencies) and binary logistic regression analysis using SPSS version 21 (SPSS Inc., Cary, SC). The comorbidities were filtered according to a possible pathophysiological hypothesis defined by the authors and according to a limited amount of inclusion of independent variables. For these reasons we included no more than 10 independent variables in the binary logistic regression. Moreover comorbidities with less than 25 cases were omitted. For every logistic regression we report the Nagelkerke R Square and Hosmer and Lemeshow test value. P-values below 0.05 were considered to indicate statistical significance.

## Results

Our initial SAH cohort consisted of 250 patients. In 46 patients suffering from SAH no aneurysm was detectable. We excluded one patient because of refused consent to participate in this study. The remaining 203 cases of aneurysmal SAH between 2012 and 2017 formed the final study cohort.

### Baseline Data

70 cases were allocated to microsurgical clipping and 133 cases to endovascular treatment. 126 (62%) patients were female. Mean age in our collective was 55.1 years (16–88 years; SD 13.4). The mean maximum aneurysm diameter was 5.9 mm (2–22 mm; SD 3.1). Most patients were attributed to “good” WFNS grades (WFNS Grade I-II: 109 patients; 53.7%), followed by “poor” WFNS grades (WFNS grade IV–V: 75 patients; 36.9%) and “intermediate” WFNS grades (WFNS grade III: 19 patients; 9.4%). The most frequent aneurysm locations were the anterior cerebral artery and the anterior communicating artery (ACA/ACoA: 85; 41.9%). Almost equal numbers of aneurysms were located on the internal carotid artery (ICA: 45; 22.2%) and the middle cerebral artery (52; 25.6%). The smallest group were aneurysms of the posterior circulation (Vertebral artery VA/ Basilar artery BA: 21; 10.3%). For an overview of the baseline data see Table 1.

**Table 1:** Baseline Characteristics.

Intervention	n	Percentage
Clipping	70	34.5%
Coiling	133	65.5%
**Sex**		
male	77	37.9%
female	126	62.1%
	**Mean**	**Standard deviation**
Age	55.1 y (16–88 y)	13.4
Mean aneurysm size	5.9 mm (2–22 mm)	3.1
**WFNS Grade**	**n**	**Percentage**
Grade I–II	109	53.7%
Grade III	19	9.4%
Grade IV–V	75	36.9%
**Location of aneurysm**	**n**	**Percentage**
ACA/AcoA	85	41.9%
ICA	45	22.2%
MCA	52	25.6%
VA/BA	21	10.3%

### Outcome and Cerebral Ischemia

During the hospital stay 39 of 203 patients suffered from early cerebral ischemia (19.2%), and 36 of 203 patients developed a DCI (17.7%).

Outcome data after one year were available in 199 of 203 patients. With a dichotomized distribution of the outcome (“good outcome” after one year: mRS = 0–2 and “poor outcome” after one year: mRS = 3–6) 55.7% of the patients had a favorable outcome and 42.4% suffered from a poor outcome after one year. 2% of the patients (n = 4) were not available for the follow up telephone interview (Table 2).

**Table 2:** Outcome data measured by modified Rankin Scale (mRS).

mRS after 1 year	n	
0 (no symptoms)	90	44.3%
1 (Minor symptoms)	12	5.9%
2 (Some restriction in lifestyle)	11	5.4%
3 (Significant restriction in lifestyle)	17	8.4%
4 (Partly dependent)	10	4.9%
5 (Fully dependent)	9	4.4%
6 (Dead)	50	24.6%
Good outcome (mRS 0–2)	113	55.7%
Poor outcome mRS (3–6)	86	42.4%
Total	199	98.0%
Missing	4	2.0%

### Comorbidities and Smoking

In our collective 69% (140 patients) had hypertension as comorbidity and 35% (71 patients) a history of smoking. But also hypercholesterolemia, hypothyroidism and diabetes mellitus were frequent (23.2%, 19.7%, 13.8% respectively). Table 3 gives an overview of the comorbidities.

**Table 3:** Comorbidities and history of smoking.

Comorbidities	n	%
Stroke	2	1.0%
Coronary heart disease	8	3.9%
Atrial fibrillation	10	4.9%
Hypertension	140	69.0%
Renal insufficiency	3	1.5%
Diabetes mellitus	28	13.8%
Ethanol abuse	16	7.9%
Tumor history	14	6.9%
Hypothyreosis	40	19.7%
Depression	33	16.3%
History of smoking	71	35.0%
Hypercholesterolemia	47	23.2%
Migraine	8	3.9%
Total	203	100.0%

### Predictors of Outcome after One Year

We performed binary logistic regression analysis in order to identify potential effectors associated with the outcome after one year. Therefore we dichotomized outcome parameters and tested with baseline characteristics (sex, age, WFNS grade), ischemia data (ECI, DCI), comorbidities (hypertension, diabetes mellitus, hypercholesterolemia, hypothyroidism) and history of smoking data. The Nagelkerke R Square value (0.67) as well as the Hosmer and Lemeshow tests (0.30) showed feasibility of the test. For the results of this analysis please see Table 4. Four patients (2%) of 203 were missing for this analysis.

**Table 4:** Predictors of outcome after one year.

Risk factors	Odds ratio	95% Confidence intervall	p
Sex	0.64	0.23–1.75	0.38
Age	1.05	1.01–1.09	0.0092
WFNS Grade	6.28	3.63–10.84	<0.0001
Early cerebral ischemia	10.06	2.87–35.35	0.00032
Delayed cerebral ischemia	4.03	1.28–12.69	0.017
Hypertension	0.18	0.062–0.53	0.0019
Diabetes mellitus	1.86	0.52–6.64	0.34
History of smoking	0.21	0.074–0.59	0.0031
Hypercholesterolemia	1.17	0.39–3.50	0.78
Hypothyreosis	0.90	0.28–2.92	0.86

Binary logistic regression was performed with dichotomized outcome (0 = “good outcome”; 1 = “poor outcome”) as the dependent variable and sex, age, WFNS grade, ECI, DCI, hypertension, diabetes mellitus, smoking history, hypercholesterolemia and hypothyreosis as independent variables.

Smoking (odds ratio: 0.21; p = 0.0031) and hypertension (odds ratio: 0.18; p = 0.0019) were predictors of good clinical outcome after one year. Age (odds ratio: 1.05; p = 0.0092), WFNS Grade (odds ratio: 6.28; p < 0.0001), early cerebral ischemia (odds ratio: 10.06; p = 0.00032) and delayed cerebral ischemia (odds ratio: 4.03; p = 0.017) were predictors of poor outcome after one year. Age had a mild influence on outcome, while early cerebral ischemia had a strong impact on the outcome after one year. But also delayed cerebral ischemia and the WFNS grade influenced the outcome significantly. Sex, diabetes mellitus, hypercholesterolemia and hypothyroidism had no significant association with the outcome after one year (p > 0.05).

### Predictors of Death after One Year

We tested the same variables as before for correlation with death after one year using binary logistic regression. The Nagelkerke R Square value (0.53) as well as the Hosmer and Lemeshow tests (0.93) showed feasibility of the test. For the results of this analysis please see Table 5. For this investigation four patients (2%) of 203 were missing. Age was a mild (odds ratio: 1.09; p < 0.0001) and WFNS grade a strong (odds ratio: 3.23; p < 0.0001) predictor for death after one year. ECI and DCI were not significant predictors (p > 0.05) as well as diabetes mellitus, hypercholesterolemia and hypothyroidism (p > 0.05). Hypertension (odds ratio: 0.12; p < 0.0001) and smoking (odds ratio: 0.31; p = 0.048) were significantly associated with the prevention of death after 1 year.

**Table 5:** Predictors of death after one year.

Risk factors	Odds ratio	95% Confidence interval	p
Sex	0.89	0.35–2.27	0.80
Age	1.09	1,049–1,141	<0.0001
WFNS Grade	3.23	1.89–5.53	<0.0001
Early cerebral ischemia	1.73	0.63–4.77	0.29
Delayed cerebral ischemia	0.76	0.26–2.27	0.62
Hypertension	0.12	0.041–0.33	<0.0001
Diabetes mellitus	1.07	0.32–3.61	0.92
History of smoking	0.31	0.098–0.99	0.048
Hypercholesterolemia	0.77	0.22–2.68	0.69
Hypothyreosis	0.36	0.109–1.20	0.097

Binary logistic regression was performed with dichotomized status of death (0 = “alive”; 1 = “dead”) as the dependent variable and sex, age, WFNS grade, ECI, DCI, hypertension, diabetes mellitus, smoking history, hypercholesterolemia and hypothyreosis as independent variables.

### Predictors of Delayed Cerebral Ischemia

We conducted a further binary logistic regression with the intension to find predictors of delayed cerebral ischemia. The Nagelkerke R Square value was 0.28 and the Hosmer and Lemeshow tests value was 0.61. For the results of this analysis please see Table 6. For this investigation all patients (n = 203) were available. Only vasospasm detected by transcranial-doppler was a significant predictor of DCI (odds ratio: 39.57; p < 0.0001).

**Table 6:** Predictors of DCI during hospital stay.

Independent variables	Odds ratio	95% Confidence intervall	p
Sex	1.56	0.65–3.78	0.32
Age	1.01	0.98–1.05	0.53
WFNS Grade	1.48	0.94–2.32	0.089
Hypertension	0.77	0.32–1.85	0.56
Diabetes	0.90	0.28–2.87	0.86
Smoking	0.47	0.18–1.18	0.11
Cholesterol	2.71	0.96–7.67	0.06
Hypothyreosis	1.01	0.35–2.88	0.99
Vasospasm	39.57	5.01–312.70	0.00049

Binary logistic regression was performed with dichotomized status of DCI (0 = “no”; 1 = “yes”) as the dependent variable and sex, age, WFNS grade, hypertension, diabetes mellitus, history of smoking, hypercholesterolemia, hypothyreosis and vasospasm detected via transcranial doppler as independent variables.

## Discussion

Contrary to our expectation we found that a history of smoking and hypertension protected patients with aneurysmal SAH from poor outcome in our cohort. In the literature the impact of a history of smoking on the outcome after SAH remains unclear, as several studies report contradictory results^9–12^. Krishnamurthy found in 320 patients that the influence of smoking on the occurrence of delayed neurological deterioration was significant but smoking did not prove to be an independent predictor of clinical outcome regardless of dose or duration^9^. Pobereskin *et al*. reported on 800 SAH cases that the relative risk of death at all time intervals was lower for smokers than for non-smokers. For the comorbidity of hypertension prior to the appearance of SAH there was no significant association found regarding the risk of mortality^10^. Recently Dasenbrock reported, that smokers had significantly decreased adjusted odds regarding tracheostomy or gastrostomy placement, discharge to institutional care and regarding poor outcome compared with nonsmokers^11^. In our single-center analysis we also found paradoxical superior outcomes for smokers.

Our results raise the question, what pathomechanism could explain a protective effect of hypertension and smoking. It was suggested before, that immediate vasospasm after the aneurysm rupture leads to a reduced severity of the initial hemorrhage in smokers^10^. Our own data can neither support nor refute this theory.

But protective effects of smoking are also known in stroke patients treated with tissue plasminogen activator (tPA)^16^. Preconditioning and adaptive cellular responses of the brain tissue associated with raised levels of carbon monoxide might play a role by a lower sensibility towards perfusion deficits in vasospasm after SAH^16–18^.

Owing to the mono-center design of this study generalizability is limited.

The association of smoking and hypertension with the incidence of vasospasm is unclear in the literature^19,20^. Hypertension and smoking might have a protective effect on the incidence of vasospasm. Since both promote atherosclerosis, an inhibited vascular reactivity could reduce the incidence of vasospasm and DCI. Also hypercholesterolemia and diabetes mellitus could hypothetically this effect. Therefore we tested our cohort regarding the association of vasospasm and DCI, sex, age, WFNS grade, hypertension, diabetes mellitus, hypercholesterolemia, hypothyroidism and history of smoking. We did not find significant associations with the occurrence of vasospasm in TCD. Moreover testing hypertension, diabetes mellitus, hypercholesterolemia, vasospasm and history of smoking as predictors of DCI did not support this theory (Table 6). Thus the protective mechanism of hypertension and smoking on outcome and death at one year remains unclear in our data as well as in previous studies regarding these theoretical pathological mechanisms involving vasospasm and DCI^19^.

Baseline characteristics have been broadly analyzed regarding their impact on the outcome after aneurysmal SAH^8^. Increasing age and neurological grade are regarded to be associated with unfavorable outcome^8^. Rosengart reports with descending importance cerebral infarction, neurological grade, age, vasospasm and history of hypertension as important factors determining poor outcome 3 months after SAH^21^. Prophylactically induced hypertension was associated with a lower risk of unfavorable outcome^21^. Similarly in our cohort (with descending odds ratios) early cerebral ischemia, WFNS Grade, delayed cerebral ischemia and age were predictors of poor outcome after one year. For death after one year, the WFNS grade had a strong and age a mild association in our cohort. Surprisingly ECI and DCI did not have a significant association regarding the prediction of death after one year.

Over the last decades case fatality of aneurysmal SAH remains high worldwide, although mortality rates have declined in industrialized nations^22,23^. The median mortality rate is denoted to be between 27% to 44% in USA, Japan and Europe^23^. Regarding the outcome of survivors, rates of persistent dependence between 8% and 20% have been reported which is comparable to our results (mRS 4 and 5 = 9.3%)^23,24^.

In our study a high proportion of very good and very poor clinical outcome is noticeable after one year. While 44.3% of the patients were mRS = 0, 24.6% had died after one year. The mortality rate of our cohort is inside the data of the published literature^23,25^. In comparison to the data of Nieuwkamp *et al*. we strictly excluded all patients who had not received treatment of the ruptured intracranial aneurysm as in the ISAT trial^23,25^.

Regarding age, sex and aneurysm size there is no significant difference of our data compared to ISAT data. In our cohort were fewer ACA/AcoA-aneurysms and ICA-aneurysms but ISAT had a lower number of MCA aneurysms and aneurysms of the posterior circulation than our collective^25^. Moreover in ISAT the randomized data included a major proportion of patients (88%) with WFNS Grade I or II in comparison to only 53.7% in our collective^25^.

Compared to ISAT the differences regarding the mortality rate and the poor outcome after one year might be explained by the higher proportion of poor WFNS grades and by a higher proportion of posterior circulation aneurysms in our collective which are known to be associated with a poor clinical outcome and mortality^8,25,26^. Interestingly the proportion of patients with a very good outcome (mRS = 0) is much higher in our cohort than in ISAT (44.3% versus 25.8% for endovascular treatment and 19.2% for microsurgical treatment)^25^.

### Limitations

A major limitation of our study is that parts of the data were retrospectively collected, including data on the history of smoking. For this reason a more detailed analysis of nicotine abuse was not possible. No information was available on the number of cigarettes per day or pack years. This is an analysis of consecutive cases of a single center in a predefined period. No power analysis was performed to define the case number. TCD examinations are known to be examiner-dependent as far as quality and reliability are concerned. Those were not conducted following a trial protocol but as part of the daily clinical routine and might therefore be of limited reliability. Acquisition of follow up data was done by telephone interview which is potentially less reliable than physical neurological examinations.

Further prospective trials powered with high patient numbers will be necessary to confirm our results. This monocenter study was not registered in a public database for clinical trials.

## Conclusions

With this study we are able to present smoking and hypertension prior to SAH as predictors regarding a good clinical outcome. This finding is in contrast to the risk elevation caused by these parameters for the occurrence of aneurysmal SAH.

## Data Availability

All data generated or analyzed during this study are included in this published article.
